# Validation of the Modena bleeding score in endoscopic sinus surgery

**DOI:** 10.1016/j.bjorl.2020.08.006

**Published:** 2020-09-30

**Authors:** Matteo Alicandri-Ciufelli, Luca Pingani, Francesco Maccarrone, Lukas Anschuetz, Davide Mariano, Gian Maria Galeazzi, Livio Presutti, Giulia Molinari

**Affiliations:** a University Hospital of Modena, Otolaryngology-Head and Neck Surgery Department, Modena, Italy; b Azienda USL-IRCCS di Reggio Emilia, HR Department, Reggio Emilia, Italy; c University of Modena and Reggio Emilia, Department of Biomedical, Metabolic and Neural Sciences, Modena, Italy; d Inselspital, University of Bern, Department of Otorhinolaryngology, Head and Neck Surgery, Bern, Switzerland; e Azienda USL di Modena, Department of Mental Health and Pathological Addiction, Modena, Italy

**Keywords:** Bleeding, Endoscopic surgery, Modena bleeding score, Sinus surgery, Surgical field rating

## Abstract

**Introduction:**

The Modena bleeding score is a categorical rating scale that allows the assessment of the surgical field in relation to bleeding during endoscopic surgery. It has recently been presented and validated in the field of endoscopic ear surgery by the present authors. The Modena bleeding score provides five grades for rating the surgical field during endoscopic procedures (from grade 1 − no bleeding to grade 5 − bleeding that prevents every surgical procedure except those dedicated to bleeding control).

**Objective:**

The aim of this study was to validate the Modena bleeding score in the setting of endoscopic sinus surgery.

**Methods:**

Fifteen three-minute videos of endoscopic sinus surgery procedures (each containing three bleeding situations) were evaluated by 15 specialists, using the Modena bleeding score. Intra and inter-rater reliability were assessed, and the clinical validity of the Modena bleeding score was calculated using a referent standard.

**Results:**

The data analysis showed an intra-rater reliability ranging from 0.6336 to 0.861. The inter-rater reliability ranged from 0.676 to 0.844. The clinical validity was α = 0.70; confidence limits: 0.64 − 0.75, corresponding to substantial agreement.

**Conclusion:**

The Modena bleeding score is an effective method to score bleeding during endoscopic sinus surgery. Its application in future research could facilitate the performance and efficacy assessment of surgical techniques, materials or devices aimed to bleeding control during endoscopic sinus surgery.

## Introduction

(ESS) is the current standard treatment for a variety of conditions affecting the nasal cavity and the paranasal sinuses, such as chronic rhinosinusitis, benign and malignant tumors or cerebrospinal fluid leaks.^1^

Being mostly a one-handed technique, ESS does not allow simultaneous use of operative instruments and blood suction, thus endonasal bleeding control represents a challenging issue for the operating surgeon.

Such narrow and highly vascularized cavities like the nasal fossae and paranasal spaces can be entirely filled with blood within few seconds, especially if the mucosa is severely inflamed as a consequence of rhinosinusitis.

Bleeding is possibly the most relevant factor that could impair the quality of the surgical field during endoscopic procedures. It has been proven that uncontrolled bleeding during endoscopic sinus surgical procedures determines poor visualization of the anatomical landmarks, prolongs surgical time and carries a higher rate of complications.^2^, ^3^, ^4^

Several techniques to control intraoperative bleeding and improve surgical view during sinus surgery (e.g. topical vasoconstrictors, total intravenous anesthesia, controlled hypotension) have been described and analysed to determine their efficacy.^5^, ^6^ These types of studies, however, are complex and prone to bias, partially because standardized and validated methods of quantifying bleeding or grading the surgical field in endoscopic view are lacking.

Among the most cited grading system is the Fromme-Boezaart grading scale, a six-point scale based on the frequency of suctioning required to maintain the clarity of the surgical field.^7^ It was validated by Athanasiadis et al. and, to present authors knowledge, it is the only bleeding score currently validated in sinus surgery.^8^ The rating in the Fromme-Boezaart grading scale depends on frequency of suctioning: this is actually a major limitation, because suctioning during ESS is also used to help to dissect and remove irrigation fluid used for clarification of the field. Consequently, the frequency of suctioning is not always proportional to the actual entity of bleeding. Another rating scale developed for ESS is the Wormald surgical field grading scale, a 11-grade scale based on number of oozing points in the surgical field and on the seconds blood takes to fill the sphenoid sinus, which makes this scale strictly dependant on this anatomical site.^8^

The Modena bleeding score (MBS) is a categorical rating scale that allows the assessment of the surgical field in relation to bleeding during endoscopic surgery. It has recently been presented and validated in the field of endoscopic ear surgery by the present authors.^9^ Being independent from a specific anatomical district or dedicated instrumentation, its application could be extended to other surgical fields, making it a potentially universal bleeding score.

The aim of the present paper was to validate the MBS in the context of ESS. A uniform and validated bleeding score like the MBS would be a reliable tool in the performance and efficacy assessment of materials and techniques used to control intraoperative bleeding in ESS.

## Methods

### The Modena bleeding score (MBS)

The MBS is a categorical scale written in English that provides five different levels (from “Grade 1 − no bleeding” to “Grade 5 − bleeding that prevents every surgical procedure except those dedicated to bleeding control”), as shown in Table 1. Being already assessed,^9^ the face validity of the MBS was not repeated for this study.

**Table 1:** Modena bleeding score.

	Scoring
No bleeding	1
Bleeding easily controlled by suctioning, washing or packing without any significant modification or slowing of surgical procedure	2
Bleeding slowing surgical procedure	3
Most of the maneuvers dedicated to bleeding control	4
Bleeding that prevents every surgical procedure except those dedicated to bleeding control	5

### Intra-rater and inter-rater reliability

After informed consent, fifteen surgeons currently working at the Department of Otorhinolaryngology-Head and Neck Surgery of the University Hospital of Modena were involved in the study as evaluators for intra-rater and inter-rater reliability assessment. 5 out of 15 of the raters perform more than 50 nasal surgeries a year. 5 out of 15 perform between 30–50 procedures a year, and 5 out of 15 perform less than 30 procedures a year.

Fifteen videos of various endoscopic sinus surgical procedures were randomly selected by one of the authors (DM) from the departmental archive of operative video recordings. Three bleeding situations (referred to as t0, t1 and t2) were selected haphazardly by the same author from each video, and then these one-minute clips were edited to produce a final three-minute video to be evaluated. Each participant had to evaluate the same randomly selected three-minute video twice, at 15-day distance using the MBS, for the measurement of intra-rater reliability. Each evaluator was also asked to assess two other edited videos from the selection, using the MBS. These evaluations were subsequently compared to those of the other evaluators on the same edited videos, to calculate the inter-rater reliability.

Intra-rater reliability was calculated using Spearman’s rank correlation coefficient ranging from -1 (perfect negative correlation) to 1 (perfect positive correlation): the strength of the correlation was defined using the following criteria: 0.00–0.19 “very weak”, 0.20–0.39 “weak”, 0.40–0.59 “moderate”, 0.60–0.79 “strong” and 0.80–1.0 “very strong”. Intraclass correlation coefficient was used for calculating inter-rater reliability (less than 0.40: poor; between 0.40 and 0.59: fair; between 0.60 and 0.74: good; between 0.75 and 1.00, excellent).^10^, ^11^

### Clinical validity

The clinical validity of the MBS was calculated using a gold standard. A group of four medical specialists in otorhinolaryngology (not involved in other areas of this study) collegially viewed and evaluated all the 45 bleeding situations present in the 15 edited videos. After extensive discussion, the group defined a unanimous score through the MBS for each bleeding situations (to be referred to as referent standard). The referent standard evaluations were then compared with those obtained for inter-rater reliability. The agreement level was calculated through Krippendorff's Alpha (<0 no agreement; 0–0.20 slight agreement; 0.21–0.40 fair agreement; 0.41–0.60 moderate agreement; 0.61–0.80 substantial agreement; 0.81–1 perfect agreement).^12^, ^13^

Considering that the agreement level was assessed on a total number of 45 bleeding situations and defining 1-β = 0.700 with a statistical significance (p) of 0.05, a sample size of 15 videos to include in this study was considered appropriate.^14^

Due to the nature of this study, it was granted an exemption by the Institutional Review Board of the University Hospital of Modena, Italy.

## Results

### Intra-rater and inter-rater reliability

As illustrated in Table 2, Spearman’s rank correlation coefficients were all above 0.600 (ranging from 0.6336 to 0.861) for intra-rater reliability, presenting an increasing rate from T0 to T2, and were statistically significant (*p* <  0.05) for all three evaluations (t0, t1, t2). The inter-rater reliability was good to excellent as the interclass correlation coefficients were equal or higher than 0.676 for the three assessments (Table 3).

**Table 2:** Intra-rater reliability.

		First video view		
Second video view		t0	t1	t2
	t0	ϱ = 0,636		
		*p* = 0,011		
	t1		ϱ = 0,768	
			*p* = 0,001	
	t2			ϱ = 0,861
				*p* < 0,001

**Table 3:** Inter-rater reliability.

		Mean intraclass correlation	95% confidence interval	
			Lower bound	Upper bound
t0	Single measures	0.844	0.671	0.941
t1		0.862	0.709	0.947
t2		0,676	0.316	0.876

### Clinical validity

The clinical validity of the MBS was α = 0.70; confidence limits: 0.64–0.75, corresponding to substantial agreement.

## Discussion

A clear visualization of the surgical field is a fundamental requirement during ESS. A small amount of bleeding can impair the surgical field and the surgeon’s ability to visualize anatomical landmarks, representing an important cause of iatrogenic morbidity (including vessels or nerve damages and cerebrospinal fluid leakage).^15^ Data from a preliminary extensive literature review performed by the present authors show that the methods used to quantify the amount of bleeding during surgical procedures could be grouped into two categories. Firstly, objective methods are based on the entity of blood lost during surgery, such as measurement of the volume of suctioned fluids or comparison between preoperative hemoglobin (Hb) in a patient’s blood and the concentration of Hb in the suction unit at the end of surgery.^16^, ^17^ Despite using quantifiable parameters, these methods usually imply specific tools and laboratories for analysis, which could be time-consuming, expensive and not easily accessible for immediate use. Furthermore, neither the effect of irrigation solution on blood nor the blood ingested by the patient are considered in these evaluations.

Secondly, subjective methods for scoring bleeding during surgery typically rely on a specific visual rating scale used by a rater, who is asked, during or immediately after the surgical procedure, to assess the bleeding amount, or more commonly, the effect of bleeding on the surgical view. The assessment is made through a defined scoring system. The most relevant advantages of these methods are their dynamicity and the direct evaluation of how bleeding could impair surgery, despite the actual quantity of blood loss. Among these, several are numerical, using either a 0–10 or 1–10 Visual Analog Scale (VAS) or defining a numerical stratification, through descriptive sentences. Numerical scores make the statistical management of data easier, compared to descriptive scores. However, a plain number lacks straightforward meaning. To overcome this, some authors have defined descriptive categories, which encompass two or three numerical scores. For example, in the study of Van Montfoort et al., given a NRS (numerical rating scale) of 0–10, with 0 defining the worst visual clarity and 10 the best visual clarity possible, an NRS was considered “poor” when less than 4, “fair” when 4 < NRS < 7, and “good” when NRS > 7. The cut-off value for the NRS was set at > 7 because, according to those authors, this was considered to represent “good intraoperative visibility”.^18^

Another concern about bleeding scores in surgery is whether the difference from one grade to another in a given system corresponds to the so-called “minimum clinically significant difference” (MCSD) of bleeding assessment or surgical field condition. As recognized by some authors, the MCSD has not been established for all scoring systems and this might lower the effectiveness of the score in assessing the real situation.^19^ In ESS, as well as in endoscopic ear surgery, it is possible that even different amounts of bleeding would similarly impact the endoscopic management, depending on the phase of the surgery and on the specific anatomical region. Indeed, the real difference between bleeding conditions lies in how bleeding affects the surgeon’s work, in terms of being irrelevant for the continuation of surgery, slowing the surgical procedure or interrupting the surgical steps at all.

In light of the above-mentioned critical aspects on bleeding rating and the lack of validated bleeding scores emerged from the literature, the authors decided to introduce the Modena Bleeding Score, that despite being a subjective method, it uniquely assesses the direct impact of bleeding on the surgical steps. Its independence from a specific instrument or anatomical structure makes it different from other scoring systems used in ESS.

From the present validation study, encouraging results for both intra-rater and inter-rater reliability were found, similarly from the analysis performed in the context of endoscopic ear surgery. The intra-rater reliability ranged from 0.6336 to 0.861, while the inter-rater reliability was between 0.676 and 0.844. The evaluations on the same video by a given rater may be more precise as the rater becomes more confident with the use of the scale in the following evaluations. This may be the reason for the increasing values of intra-rater reliability from t0 to t2 found in this study.

The comparison between inter-rater reliability and the referent standard (referred to the group that collegially evaluated all videos during clinical validity phase) produced a Krippendorff's Alpha score of 0.70, corresponding to substantial agreement.

Surgical field conditions may change several times during a single procedure; so theoretically, a good scoring system should also convey the concept of time. Trying to achieve this aim, Wu and colleagues assessed the visual field (during upper gastro-intestinal endoscopic evaluation of bleeding) before and after irrigation with saline and H_2_O_2_. The images were scored as a worsening or improvement in the field, using a “visual clearance” scoring system: -3, marked worsening of visual field; −2, moderate worsening; −1, slight worsening; 0, no change; +1, slight improvement; +2, moderate improvement; +3, marked improvement.^20^

Regarding the MBS, the sense of time is not included in the score system itself, though it has been developed to be a easy-to-use and fast tool, ideally applicable any time the rater has the impression that the bleeding condition is changing during surgery. Considering all the ratings from a single surgery and the surgical time, a linear chart could graphically describe the variability of bleeding conditions during the surgical procedure.

Another possible way to apply the MBS, similar to other bleeding scores, is to pre-operatively set a time range by which the surgeon has to repeatedly rate the surgical conditions. For example, Little et al. applied the Wormald and the Boeazaart grading scales at regular 15 min intervals to assess the impact of total intravenous anesthesia versus inhaled anesthetic during endoscopic sinus surgeries.^21^ This standardized method could facilitate the comparison among different surgeries of similar duration. Overall, the simplicity of the MBS makes it a dynamic instrument whose use could be standardized according to the setting’s requests.

## Conclusion

In the opinion of the present authors’, the MBS represents a valuable tool, easily applicable during the surgical intervention as frequently as the surgeon feels that there is a change in how the intraoperative bleeding is influencing his or her endoscopic work. Considering the hallmarks of the MBS as compared to other bleeding scores and the results of this validation study, it represents a reliable tool to assess the bleeding conditions during endoscopic sinus surgical procedures. The MBS may become the standard method to assess the performance and efficacy of hemostatic materials and techniques used to control intraoperative bleeding in ESS.

## Conflicts of interest

The authors declare no conflicts of interest.
